# Benefits, barriers and enablers of mentoring female health academics: An integrative review

**DOI:** 10.1371/journal.pone.0215319

**Published:** 2019-04-18

**Authors:** Merylin Cross, Simone Lee, Heather Bridgman, Deependra Kaji Thapa, Michelle Cleary, Rachel Kornhaber

**Affiliations:** 1 Centre for Rural Health, School of Health Sciences, University of Tasmania, Australia Launceston, Tasmania, Australia; 2 College of Health and Medicine, University of Tasmania, Australia Alexandria, NSW, Australia; Aga Khan University, KENYA

## Abstract

This integrative literature review synthesizes the primary research evidence on mentoring female health academics published from 2000 to 2018, to identify the benefits, enablers and barriers to mentoring women. The need for this review is underpinned by the magnitude of change in higher education, the high number of women in health disciplines, limited progress in advancing women’s academic careers, escalating role expectations, faculty shortages and staff turnover. Data were sourced from Scopus, PubMed, EMBASE and Cumulative Index of Nursing and Allied Health Literature. Twenty-seven studies were included. Although effective mentoring facilitates personal and career development, academic craftsmanship, psychosocial support and job satisfaction, it is complicated by organizational factors and personal and relational dynamics. Enablers of mentoring are mentor availability and expertise, supportive relationships, mutuality and responsiveness. Lack of, or inadequate mentoring compromise women’s job satisfaction, career development and academic productivity. Providing female health academics access to experienced, well-connected mentors with common interests who are committed to advancing their career, is an investment in optimizing potential, promoting supportive work environments and increasing productivity and retention. Realizing the institutional potential that mentoring female health academics offers, is contingent on academic leaders valuing mentorship as faculty business and understanding the role that the contemporary academic environment plays in achieving mentoring outcomes. Further empirical and longitudinal research is needed to evaluate effective approaches for mentoring women in the contemporary academic environment.

## Introduction

Academic mentorship features prominently in orientation, support of new faculty transitioning to the academic role, faculty development, career advancement, job satisfaction and retention [1–4]. However, the reforms and turnover in higher education over the past two decades [5–7] have made mentoring more challenging, particularly for women. The restructuring of the academic workforce and intensification of academic work [5, 8–10] have resulted in lower job satisfaction, increased turnover and faculty shortages [11–13]. The limited mentorship available to female academics is compounded by the continuing lack of female representation in senior academia and ageing of the professoriate [14–16]. These changes have implications for providing mentorship, particularly in faculties of health where a number of disciplines such as nursing, psychology, physiotherapy, pharmacy, and occupational therapy, are predominantly female [11, 13, 17].

The roots of mentoring lie in Greek mythology where a mentor was considered a sage and trusted counsellor [18, 19]. Traditionally in higher education, mentorship has been seen as a long-term mutually beneficial relationship between a junior and senior academic [3, 4]. While much attention has been given to traditional dyadic mentoring and the attributes of good mentors, scant attention has been given to the shifting academic environment and its influence on mentoring outcomes.

Recent changes in higher education have spawned alternative forms of mentoring such as collegial, facilitated peer, functional, online and distance mentoring [2, 20, 21]. A panel of medical academic experts in the USA concerned with the lack of conceptual clarity around mentoring, re-conceptualized it as a construct:

…that may vary along a continuum from informal/short-term to formal/long-term in which faculty with useful experience, knowledge, skills, and/or wisdom offers advice, information, guidance, support, or opportunity to another faculty member or student for that individual’s professional development (p. 67) [19].

This more flexible conceptualization of mentoring reflects efforts to adapt to the restructured higher education environment which has become a corporatized global knowledge industry [6, 7, 22]. The legacies of corporatization have been casualization of the workforce, demanding workloads, declining government funding and pressure on academics to meet escalating teaching and research performance expectations [5, 8–10, 22]. Female health academics have been particularly vulnerable to casualization [13, 15, 16]. Reliance on part-time, short-term sessional or adjunct positions, have eroded working conditions and job satisfaction, created unprecedented job insecurity and led to attrition [9, 11, 23, 24].

Despite significant attention to advancing women’s careers in academic medicine, only ‘modest’ progress has been achieved [2, 25]. As with other faculties, obstacles to women’s academic advancement have included organizational barriers, staff turnover, gendered roles, family responsibilities [14, 16, 25, 26] and double standards [26]. Mentorship, though often lacking [2–4], has been proposed as a solution [1, 27]. The need for mentorship is further justified by the lack of qualified faculty and urgent need to recruit and retain new staff [2, 4, 13, 28].

In a systematic review of mentorship in academic medicine, Sambunjak et al. [4, 29] highlighted a lack of clarity about the effectiveness of strategies to enhance mentoring for women and the impact of gender on the mentoring dynamic. For this review, our aim was to synthesize the evidence available on the provision of mentoring for female health academics, identify the benefits, enablers and barriers to mentoring women, gaps in knowledge and the consequences of a lack of, or inadequate mentorship.

## Methods

### Review process

We adopted an integrative review process based on the five-stage process proposed by Whittemore and Knafl [30]: developing the review question, searching the literature, data collection, discussion of results and presentation of integrated findings. The integrative approach was chosen because it accommodates different methodologies and levels of evidence and provides a rigorous approach that is conducive to reviewing, analysing, and synthesising the primary research literature and generating comprehensive practical, conceptual or theoretical understanding [31, 32].

Although diversity is a key strength of the integrative review process, it renders quality appraisal somewhat problematic and limiting [30]. Whittemore and Knafl [30] argue that while issues such as methodological soundness and authenticity are important, studies should not be excluded on the grounds of quality appraisal. To capture informational value, no studies were excluded based on quality.

### Review questions

Our research questions were: What are the benefits, barriers and enablers of mentoring female health academics, the consequences of a lack of, or inadequate mentoring, and the gaps in knowledge about mentoring women?

### Literature search strategy

We conducted a systematic and rigorous search in July 2018 across the electronic databases considered to encompass a wide-ranging multidisciplinary span of research relevant to the healthcare domain: PubMed, EMBASE, CINAHL and Scopus. Boolean connectors AND, OR and NOT were used to combine search terms including mentor*, women, female, higher education, universit* and academi* (S1 Table).

### Inclusion and exclusion criteria

Our search criteria incorporated peer-reviewed primary research on the mentorship of female health academics published in English from 2000 to July 2018. Research articles on mentorship where the majority (>90%) were female participants or those reporting gendered findings were also included. We excluded articles published prior to the year 2000 because of the significant changes occurring in higher education that have influenced the need for mentoring and women’s access to mentorship. Reviews, theses, conference proceedings and editorials were excluded, as were studies involving students and clinicians.

### Data collection

**Fig 1:**
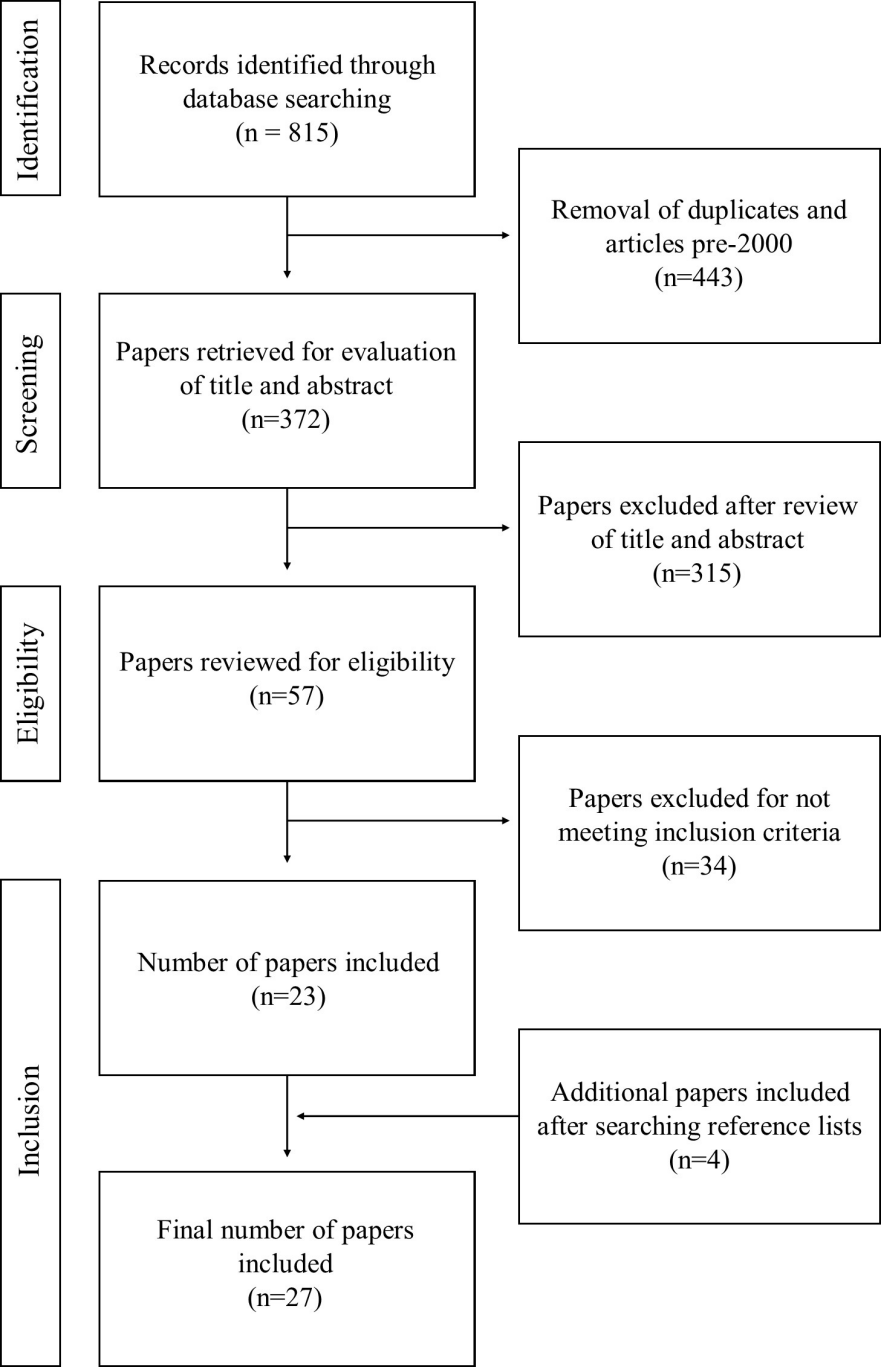
Decision trail for selecting included studies.

The database search generated 815 records. After removing duplicates, 372 potential studies were identified. Three authors independently screened the titles and abstracts of prospective papers against the inclusion criteria and identified 57 studies. In the case of disparities, consensus was achieved by examining the full-text and collaborative discussion. Through this systematic process, 34 studies were removed leaving 23 for inclusion. After scanning reference lists of included and review papers, four additional studies were identified, yielding a total of 27 (Fig 1).

### Data extraction and synthesis

A summary table was generated synthesizing the data from included studies. Data extracted included author(s), year of publication, country of origin, purpose of study, sample, design and data collection, method(s) of analysis and significant findings germane to the review aims (Table 1). Results were synthesized regardless of the level of evidence in keeping with the integrative review process which seeks to capture the breadth of evidence available [30]. To address the research questions and facilitate the synthesis of disparate data, we categorized and thematically analysed the findings to identify recurring relationships [30]. Finally, we developed a concept matrix as suggested by Torraco [32] to map thematic content to source studies.

**Table 1:** Summary of mentoring studies reviewed.

Author, year and country	Purpose	Sample and study population	Study design and data collection	Methods of analysis	Key mentoring outcomes
Athanasiou et al. [33] (2016) UK	Investigate gender disparities in research performance such as mentoring and scientific collaboration	N = 104 (34F) professors in Faculty of Medicine	Cross-sectional survey Mentoring perception survey, bibliometric analysis and social network analysis	Correlation, regression analyses and Mann-Whitney U test	No significant gender differences in mentoring skills, quality, frequency or satisfaction of mentoring and number of publications, citations and h-index.
Blood et al. [34] (2012) USA	Better understand the characteristics and components of mentoring desired by women	N = 1179F from medicine and dentistry; 5% professors, 13% associate professors, 28% assistant professors and 53% instructors Median age 44 years 83% worked full-time	Cross-sectional survey	Chi-square test, t-test, ANOVA and logistic regression	54% had a mentor, 72% without mentor indicated need for mentorship and 39% reported insufficient mentoring impacted career advancement. Important mentor characteristics: availability (71%), program development and strategic planning experience (54%), clinical experience (41%) and teaching experience (41%). Minorities more likely to consider gender and race important. <50% reported mentoring needs met such as program development, strategic planning, shifting career needs and negotiating skills. Unmet needs rated highest importance were career goal-setting and negotiation skills (52%). Lower ranked faculty interested in mentoring for career advancement and writing, while higher ranks identified need for mentoring on strategic planning. Those with children identified mentoring gaps in finding collaborators and work-life balance.
Carapinha, et al. [35] (2016) USA	Investigate the mentor characteristics women faculty in academic medicine report most important	N = 3100F women faculty in medical schools at instructor level or higher White: 68%, age ≤44 years: 48%, assistant professors: 41% and instructors: 23%	Cross-sectional survey	Chi square tests and ordered logistic regression	53% currently had a mentor, 34% had been mentored in the past, and 13% had never had a mentor. Participants identified having a mentor in the same department and institution important. Faculty of lower rank had greater preference for mentors with similar personal and career interests. Lower rank faculty, Black faculty, and those not currently mentored had a greater preference for mentors of same gender. In general, women considered having mentors of same race/ethnicity less important, except for racial/ethnic minorities, foreign-born faculty, and those who had never had a mentor.
Chung & Kowalski [36] (2012) USA	Examine mentoring relationships among nursing faculty to understand influences on job stress, psychological empowerment and job satisfaction	N = 959 nursing faculty; participants were generally female, mean age 53 years, worked full time and had a PhD	Cross-sectional survey including validated scales of mentoring, faculty stress, psychological empowerment and job satisfaction	Pearson’s correlation, t-tests and multiple linear regression	40% had mentor; 76% felt mentoring quality was good; having a mentor associated significantly with higher psychological empowerment, lower job stress and higher job satisfaction; Positive relationship among mentoring quality, psychological empowerment and job satisfaction.
Colletti et al. [37] (2000) USA	Determine if concerns expressed by male and female surgeons reflected broader concerns for academic surgery and medicine	N = 54 (9F) medical faculty Most women were from tenure track and just over half were senior faculty; none held a PhD and/or MD	Cross-sectional survey	Univariate descriptive statistics, t-tests (but t-statistic and p-value not reported)	While two in three women had a mentor; most mentors were male; Women perceived mentoring as a specific area of bias. Women received critique on clinical performance and scientific work less often than men. Most women felt their mentors actively fostered their career though 56% also reported that mentors used their work to further their own career. Only one woman (compared to 71% of men) considered there were role models in their section and department.
De Saxe Zerden et al.[38] (2015) USA	Understand the lived experience of social work female faculty regarding supports and barriers to professional development	N = 10F social work, non-tenure track faculty members Age: 34 to 56 years 100% had a masters, 1 PhD, 1 enrolled in a PhD	Qualitative phenomenological study In-depth interviews demographic questionnaire and exit surveys	Open and constant comparative coding and negative case analysis	Mentoring was the most frequently cited form of professional development. Mentoring was considered the most helpful and third most needed activity, and the lack of mentoring the fourth most common barrier to professional development. When mentoring was not available, participants felt isolated and discouraged.
Dutta et al. [39] (2011) UK	Pilot mentoring scheme for female academics; evaluate health and attitudinal benefits; compare mentor and mentee pre and post expectations and achievements	N = 46 mentoring pairs All the mentees were female from psychiatry (44 completed pre-mentoring survey, 37 at 6 months and 30 at 1 year). Rank ranged from research assistant to senior lecturer	Mixed method Quantitative: Single arm, pre-post-test (baseline, 6 months and 1 year) Qualitative: expected gain from the mentorship process	Paired t-tests, McNemar’s test, content analysis and charting	With mentoring, self-esteem, self-efficacy and job-related wellbeing improved and work–family conflict reduced at 1-year follow-up. Mentoring produced no improvement in job satisfaction. Benefits to mentees: improved confidence and assertiveness, receipt of support and encouragement, and space to reflect on career goals, pre-mentoring expectations of career progress not achieved at 1-year follow-up.
Elliott et al. [40] (2010) USA	Report how native American women in medical faculty describe personal and professional success to better inform mentoring	N = 5F Native American women academics and physicians; age range 42–60 years	Qualitative Open-ended interviews	Unified coding system, concurrent and continuous data collection and analysis until saturation	Mentoring relationships had positive impact on personal and professional success. Women described benefits of mentoring as emotional support, role modelling, problem solving, help negotiating the system, and referrals for personal matters. Mentoring was considered beneficial at beginning of career though needs shifted with life’s circumstances.
Files et al.[41] (2008) USA	Assess outcomes of a facilitated peer mentorship program for female faculty	N = 4F physician instructors	Single arm, pre-post-test (baseline, and 10 months follow-up) Self-assessment and skill acquisition survey	Descriptive comparison of pre and post-test scores	10-month follow-up found 30% improvement in satisfaction with academic accomplishments, achievement of skills needed for advancement and belief in writing skills. 3 co-authored 3 peer-reviewed manuscripts and all 4 achieved promotion.
Fleming et al. [42] (2015) USA	Explore the efficacy of a faculty development mentoring program for early career faculty	N = 104 (69F) junior medical faculty 48 completed baseline, 43 completed follow-up and 27 completed both	Single arm, pre-post-test (baseline, and 18 months follow-up)	Wilcoxin rank-sum test, Wilcoxin signed rank test, Linear regression and network analysis	Increase in self-reported knowledge, skills and attitudes in professional development and scholarship (p < .05). Female faculty demonstrated greater improvement compared to male faculty with regard to professional development (p < .05).
Foster et al. [43] (2000) USA	Determine how faculty’s perceptions of medical school gender climate differ by gender, track, rank and department	N = 507 (only 497 provided gender data -127F) faculties of medicine	Cross-sectional survey	Fisher’s exact two-tailed test	75% male and 69% female assistant professors had mentors. Both genders across all ranks indicated satisfaction with mentors on facilitating their professional development and revision of their progress. Women more likely to be mentored by men: 100% full professors, 74% associate professors and 65% assistant professors. < 5% of men had female mentors. Women were more likely to feel they were used to further their mentors’ careers.
Jeffers and Mariani [44] (2017) USA	Explore the influence of a formal mentoring program on career satisfaction of novice nurse faculty	N = 124 (118F) working as faculty for five years or less. Age: Mean 47.2 years (range 30 to 67) 47.6% master’s degrees, 32.3% PhD, and 20.2% Doctor of Nursing practice degree. 81.5% non-tenure track	Mixed method Cross-sectional survey with open-ended questions	Chi square test, t-test and content analysis	31% were mentored, and 71.8% of these found mentoring supportive and valuable. No statistically significant differences in career satisfaction scores and intent to stay. Most nurses considered transitioning from a clinical role to academia difficult and experienced frustrations working in an unfamiliar environment without adequate mentorship support. Participants without mentors and those with unhelpful mentors sought alternatives such as ‘trial and error’ (e.g. informal mentoring).
Koopman & Thiedke [45] (2005) USA	Investigate the attitudes of family medicine department chairs towards mentoring emphasising female and minority faculty	N = 13 (4F) chairs of Department of Family Medicine; years within the medical department ranged from 2–16 years; years as chair ranged from 2–22 years	Qualitative Semi-structured interviews	Thematic analysis using immersion crystallization technique and consensus	No consensus on whether women mentees should be paired with male or female mentors, though several felt that female mentees would benefit from other women’s advice. Chairs suggested multiple mentors for a female faculty member (e.g. male mentor for her interest area and female mentor for lifestyle issues). Concern for trust and vulnerability of mentee (e.g. boundary crossing in male/female pairing). Lack of senior women to mentor junior women.
Levine et al. [46] (2011) USA	Understand perspectives of female physicians who left academic medicine	N = 20F physicians who had left academic institution Faculty members for a mean of 3.3 years 8 were instructors and 12 assistant professors when they left	Qualitative Semi- structured interviews	Categorical analysis, individual and comparative coding	Poor mentoring or lack of mentorship was a key factor in women deciding to leave academic medicine. Lack of mentorship created a sense of dissatisfaction, frustration and discouragement with work and was a barrier to career advancement and productive research career. Inability to identify a committed mentor impeded research/grant activity.
Mayer et al. [47] (2014) USA	Evaluate long term impact of a facilitated peer mentoring program on academic achievements	N = 33F instructors and assistant professors from faculties of medicine participated in facilitated peer mentoring program for 1 year 16 participants completed both pre- and post-participation survey	Single arm, pre-post-test (baseline, and 1.25 to 6 years (median, 4 years) follow-up) Self-assessment survey on academic skills and career goals	Paired t-tests	Peer mentoring program showed long-term improvement in perceived mastery of academic skills, academic promotion and increased academic activity, including peer-reviewed outputs. Follow-up participants perceived program positively with 44% continuing to work with original peer mentoring group.
McGuire et al. [48] (2004) USA	Understand female physicians’ perceptions of gender discrimination and their needs for academic success	N = 163F medical faculty Mean age: 42.5 years 86% full time	Cross-sectional survey	One-way ANOVA, Tukey follow-up tests and independent t-tests	Mentoring was identified as the third most important need for female medical academics for grant preparation and career advancement.
McMains et al. [49] (2018) USA	Explore the prevalence and effects of mentorship, including whether sex differences exist among faculty at a military academic center	N = 104 (34F) academic medicine faculties of military academic institutions Internal medicine, paediatrics and surgery specialities were most common	Cross-sectional survey	Chi square test, Fisher exact test, Mann-Whitney U test, Kruskal-Wallis test, and logistic regression	42.1% of faculty reported currently having a mentor (53.1%F and 38.6%M, P = 0.17). Women were significantly less likely (27%) to receive formal mentorship at their first military station compared to men (44%), (Odds ratio 0.38; P = 0.049). Women considered mentorship helped to develop clinical skills, academic promotion, understand department/institution, clarify goals and research. No significant gender difference on perceived effectiveness of mentorship during residency training or as a new staff member.
Ramanan et al. [50] (2002) USA	Describe prevalence of mentoring in hospitals and institutions and identify specific factors associated with mentoring	N = 2131 (827F) assistant professors and instructors in academic medicine	Cross-sectional survey	Chi square and logistic regression	41% of women and 38% of men had an academic mentor. 50% of women and 53%of men were satisfied with mentorship. No significant gender difference for having a current mentor and satisfaction with mentoring. ‘Taking into account gender issues’ mentorship was equally important for women and men.
Seemann et al. [51] (2016) Canada	Explore career satisfaction and advancement for women in academic surgery	N = 81F surgeons 86% were aged 36 to 55 years Rank ranged from lecturer/instructor to professor with assistant professor (46%) most common; Years in practice ranged from 1 to > 15 years	Cross-sectional survey with open text-boxes	Descriptive statistics and thematic analysis	79% had at least one mentor; 89% of mentors were men; 95% of mentors were another surgeon; 54% wanted better mentoring. Many participants wanted more women as mentors for advice on balancing career, family life and personal goals. Lack of appropriate mentorship was the major challenge for women in academic surgery for career satisfaction and advancement.
Simon et al. [52] (2004) USA	Examine the experiences of African American women in leadership roles in social work education as protégés and mentors	N = 14F deans and directors of social work programs Age:> 40 years	Cross-sectional survey	Descriptive statistics	All participants had a mentor during their career; 50% had mentors at ages 25–30 years; 37% had mentors at 30–35 years. Mentor served career functions (challenging assignments, opportunities for exposure and visibility); and psychosocial functions (sense of caring and helpful advice). Career mentoring was considered more important than psychosocial mentoring.
Sonnad & Colletti [53] (2002) USA	Identify roles women are fulfilling in academic surgery and obstacles to their success	N = 724 (386F) academic surgeons 73% men and 44% women were senior faculty; 52% women and 61% men in tenured tracked positions	Cross-sectional survey	Descriptive statistics and t-tests	67% women and 54% men reported having a mentor; 97% men and 79% women had a male mentor; 2% men and 15% women had female mentors; 2% men and 6% women had both a male and female mentor. Men and women reported receiving equal critique for scientific and clinical work. 70% of men and women reported mentor actively fostered their career; 31% of women and 42% of men reported mentors utilised mentee’s work to advance their own career. 38% of women and 65% of men agreed there were good role models in their department.
Steele et al. [54] (2013) Canada	Explore views of junior faculty to inform mentorship program development	N = 175 (59F) junior medical faculty in clinical departments, among which 8 (4F) participated in focus groups and 19 (10F) in interview Majority (138) were assistant professors	Mixed method Cross-sectional survey, focus group and interview	Descriptive statistics, content and thematic analysis	Most female faculty reported having mentors of the opposite sex. Females identified lack of researcher role model as one of the major challenges. Females preferred mentors/role models of similar age and wanted advice on promotion and work-life balance, while males valued advice on finance and grants.
Straus et al. [55] (2009) Canada	To explore mentor–mentee relationships among people who had early career support	N = 28 (21 (4F) mentees and 7 male mentors) Mentees were population health or research clinician investigators awarded early career support	Qualitative Semi-structured interviews	Grounded theory approach using open, axial and selective coding	Male and female participants considered good mentorship vital to career success with most experiencing positive mentoring. Responses were mixed about whether there is a need for gender matching between the mentor and mentee. Female mentees identified challenge of finding mentors who could provide guidance around work-life balance and timing of maternity leave.
Turnbull & Roberts [56] (2005) Australia	Investigate the relationship of mentoring to scholarly productivity among nurse academics	N = 156 (128F) full-time nurse academics	Cross-sectional survey with opportunities to comment Stratified random sampling	Correlations, multiple regressions and thematic analysis	Significantly higher proportion of women (90%) perceived mentoring personally important compared to men (64%) (p = 0.001). Participants perceived mentoring less important as academic rank increased. The major challenges of mentoring were teaching workload and non-supportive cultural climate (non-collegial, exclusive and competitive; lack of incentives and rewards). Paucity of mentorship not confined to female academics. In female dominant professions, such as nursing, mentorship to men is even more important.
Varkey et al. [57] (2012) USA	Examine the impact of facilitated peer mentoring on scholarly output	N = 19F from department of medicine (6 assistant professors, 11 academic instructors, 1 clinician and 1 nurse) participated in one-year peer mentoring program Average years in faculty: 6.2, range 1.5–22 years;	Single arm, pre-post-test (baseline and 1 year follow-up) Self-assessment survey on academic skills, self-efficacy, and career satisfaction	Paired t-test	After 12 month mentoring program, 9 papers submitted for publication, 2 faculty pursued advanced degrees, one was promoted, and five submitted successful grant applications. There was a significant increase in satisfaction in academic achievement, academic skills, confidence, effective networking and identifying an effective mentor.
Wasserstein et al. [58] (2007) USA	Explore multiple aspects of mentoring in academic medicine in relation to faculty rank, track and gender	N = 1046 (262F) faculty from School of Medicine. 388 tenure track, 128 research track 476 assistants, 278 associates and 286 professors	Cross-sectional survey	Chi square, correlation, and logistic regression	No difference in having mentor between male and female assistant professors, but in case of associate professors, a larger proportion of women had a mentor. Among the assistant professors, females (68%) less often had men as a primary mentor compared to males (85%) (p < .0001), but among the associate professors there was no difference in mentor gender. A higher proportion of female associate professors had multiple mentors. There were no significant differences in satisfaction with mentoring between men and women or between those with a mentor of the same or different gender. Participants felt that mentors provided more advice than opportunities.
Welch et al. [59] (2012) USA	Describe content, value and ongoing achievements of a mentoring program for women in Emergency Medicine	N = 46F emergency medicine residents, faculty and alumni who participated in mentoring program from 2004 to 2010	Single arm, post-test only Post-test was conducted in 2010	Descriptive statistics and thematic analysis	87% reported mentoring program provided inspiration and guidance and 60% reported benefiting from peer-mentoring relationship. Participants identified social networking, inclusiveness, supportive nature, group camaraderie, and opportunities to connect with women with similar experiences as the best features of the mentorship program. The session on work-life balance was the most appreciated common thread for advancing women’s careers.

Note: F Female participants

## Results

### Study characteristics

Analysis of the study characteristics presented in Table 1 revealed that 21 of the 27 articles reviewed originated from the USA, three from Canada, two from the United Kingdom and one from Australia. Apart from medicine, nursing and social work were the only disciplines to address gender. Collectively, the studies reviewed drew on data from 8,055 women although only half of them focused exclusively on female academics. The studies utilised a variety of mentoring modalities including traditional dyadic mentoring of senior and junior academics, facilitated peer mentoring whereby a senior academic mentored a group of less experienced mentees, formal and informal approaches, multiple mentors, and peer, collegial and collaborative relationships. One study compared outcomes for those who were, as opposed to those who were not mentored. None compared the outcomes of different models of mentoring. Fourteen studies employed a cross-sectional survey, five utilized a descriptive qualitative approach, and three used mixed methods. Six studies, including one mixed method, used pre- and post-mentoring surveys with follow-up results from 10 months to 6 years.

### Key themes

**Fig 2:**
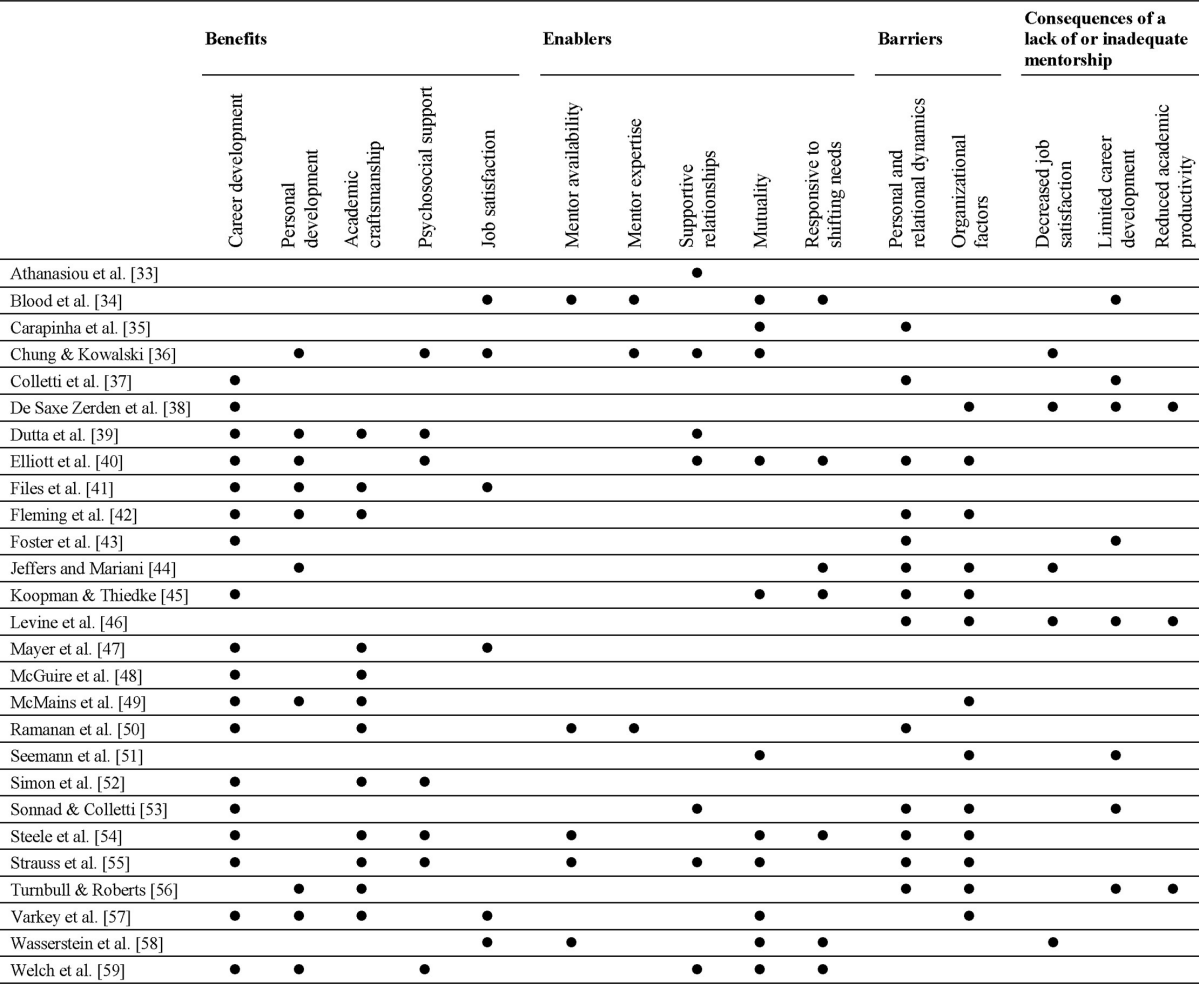
Concept matrix mapping mentoring themes to source articles.

The key themes reflect the review aims; namely, the benefits, barriers, enablers and outcomes of lack of, or inadequate mentoring for women. Thematic analysis revealed 15 major sub-themes (Fig 2). The themes pertaining to benefits were: *career development*, *personal development*, *academic craftsmanship*, *psychosocial support* and *job satisfaction*. The themes associated with barriers to mentoring were *personal and relational dynamics* and *organizational factors*. Mentoring was enabled by *mentor availability*, *mentor expertise*, *supportive relationships*, *mutuality* and *responsiveness to shifting needs*. The lack of, or inadequate mentoring for female academics led to *decreased job satisfaction*, *limited career development* and *reduced academic productivity*.

### Benefits of mentoring

#### Career development

The majority of the studies reviewed found that mentoring benefited career development by engendering valuable professional growth and fostering women’s careers. Mentorship provided a structured process for career planning and professional development [38, 42, 43, 48, 55], albeit more for men than women [37] and facilitated opportunities for exposure and visibility [52]. Mentoring enabled women to achieve academic career goals conducive to promotion and academic success [33]. Women in lower academic ranks perceived mentoring to be important for career development in general, while those in higher ranks considered it particularly beneficial in helping them strategically plan their career path [34].

#### Personal development

Mentoring provided women access to successful role models and promoted psychological empowerment and assertiveness, self-efficacy, self-esteem, confidence, job related well-being and problem-solving [39, 42, 44]. Other benefits of mentoring included space for women to reflect on and reconcile their core values with academic and personal goals, and ability to navigate multiple roles and balance work-family needs [34, 36, 40]. Women described the benefits of mentoring as improved problem solving skills, emotional support, referrals for personal matters [40] and development of clinical skills [49]. Turnbull and Roberts [56] reported a significantly higher proportion of women (90%) perceived mentoring personally important compared to men (64%) (p = 0.001).

#### Academic craftsmanship

The skills deriving from mentoring such as proficiency in academic teaching, research, writing [41], publication and grant writing [48, 57] were categorized as academic craftsmanship. Other markers attributed to craftsmanship were that mentorship facilitated scholarly productivity [33, 56], academic promotion [41, 47, 49, 57], networking and collaboration [57] and help negotiating the system [40, 44]. The facilitated peer mentorship model [41, 47, 57] increased the academic capability and publication output of junior female faculty to such an extent that many of the participants continued to work with the original peer mentoring group after completing the program [47].

#### Psychosocial support

Having a mentor fostered psychosocial support by providing encouragement, motivation, confidence, assertiveness, a sense of caring, inspiration and guidance [36, 39, 52, 59]. Additionally, mentors provided professional advocacy which facilitated social networking, inclusiveness, a supportive framework and camaraderie [39, 59].

#### Job-satisfaction

Mentoring was associated with job satisfaction, tenure and retention [36, 41, 57] though not without caveats. Dutta, et al. [39] found that although mentoring positively impacted promotion and anxiety-contentment, there was no evidence that it improved job satisfaction and attributed this to the local environment and institutional turmoil. Although Jeffers and Mariani [44] reported no significant differences in career satisfaction scores and intent to stay between those who were or were not mentored, these results should be interpreted cautiously due to the low response rate.

### Enablers of mentoring

#### Mentor availability

The availability of mentors was key to female faculty having access to mentoring [33, 35]. Availability also involved suitable mentors being willing and having time to mentor, keeping in touch regarding progress and being responsive to mentees’ needs [50, 54, 55]. Hybrid models of mentorship such as facilitated peer, group mentoring and collaborative distance mentorship effectively circumvented the lack of available senior women mentors [55, 57, 58, 60].

#### Mentor expertise

Having access to an experienced mentor with expertise in clinical practice, teaching and research facilitated role modelling [36]. Mentor expertise was also associated with strategic planning, clinical and teaching experience [34], academic guidance, professional decision-making and building professional networks [50].

#### Supportive relationship

The efficacy of mentorship was enhanced by having a supportive relationship [33, 40, 59], particularly when cultural expectations were honoured and mentees received ongoing support [40]. To be effective, mentors needed to respect and value the mentee as a person as well as a professional [36], listen to their ideas and concerns and help mentees develop their independent academic identity [55].

#### Mutuality

Repeated references to effective mentoring necessitating a certain rapport and ‘chemistry’ were reinforced by studies demonstrating that matching mentors and mentees based on mutual interest and shared understanding achieved better outcomes for both [40, 51, 54]. Whereas women, especially lower ranked faculty, preferred mentors in the same department or institution, with similar career and personal interests, those from ethnic minorities and foreign-born faculty considered having the same background important [35]. For some mentees, the sensitivity of the mentor was more important than gender [45]. While some female mentees reported having no gender preference in mentors [58, 59], others wanted female mentors who were role models at different stages of life and career who could provide advice on finding a healthy work-life balance [40, 51].

Facilitated and collaborative peer mentoring were valued for taking the power out of the mentoring relationship and fostering shared understanding. A benefit of facilitated peer mentoring was that it involved senior academics overseeing peer mentors and required limited institutional resourcing. This form of peer mentoring was valued as a successful long-term strategy to provide women access to colleagues who understood their situation, shared their academic interests and sought to progress their academic skills to achieve career goals [47, 57].

#### Responsiveness to shifting needs

One of the defining needs for female academics in the studies reviewed, was for mentors to be responsive to their shifting needs over time. The need for sameness between mentors and mentees reduced with age and experience [35]. There was recognition that ‘shifting needs’ could be addressed by multiple mentors with different skills [45]. Wasserstein, et al. [58] found that having multiple mentors achieved more than the dyadic model and related strongly to job satisfaction.

Although over two thirds of the articles reviewed provide a body of evidence supporting the value of mentoring for female health academics, there were signals that the workplace was not always conducive to mentorship or realising its potential.

### Barriers to mentoring

#### Personal and relational dynamics

Personal and relational barriers to mentoring for female faculty included the variable quality of available mentors and incongruent assignment of mentors [54, 55]. The often lower status and profile of female academics, together with the need to align personal factors and ensure a good match, limited the access female faculty had to quality mentors [40, 54]. Women often found it difficult and time-consuming to find a suitable mentor with whom they shared similar interests [46] and some men reported difficulty giving criticism to women [45]. Personal and relational dynamics were compounded for some women due to their individual attributes such as age, gender, cultural differences, past experience and fluctuating needs [40, 58].

The power differential inherent in the hierarchical structure of traditional dyadic mentoring relationships represented an important relational dynamic that sometimes rendered mentees vulnerable to exploitation [37, 45, 50, 53, 56]. Inappropriate mentor behaviour such as bullying, and incivility had a significant negative impact on mentee’s mental health and well-being [44]. Adopting a top-down approach to mentoring, without considering personal, cultural and relational factors was perceived counter-productive [40, 55]. Conversely, choice, facilitated peer, collaborative and collegial mentoring, were seen to alleviate power, vulnerability and exploitation [55].

#### Organizational factors

Organizational barriers to mentoring female faculty included the lack of mentoring available to women [38, 46, 51, 53], lack of senior women available to mentor [45], lack of mentors with specific expertise such as research [54] and shortage of mentors with mutual interests to relate to [40, 54]. Lack of time was another impediment for mentors and mentees as often they were both too busy and over-extended [46, 55, 56]. There was variable willingness to assist less experienced staff [56], a factor potentially exacerbated by the lack of institutional support; valuing of mentoring in workloads, performance expectations and promotion criteria, and incentives to mentor such as dedicated time and remuneration [55, 56].

### Consequences of a lack of or inadequate mentorship

Eleven of the 27 studies reviewed identified the consequences of a lack of, or inadequate mentoring to be decreased job satisfaction, limited career development and reduced academic productivity.

#### Decreased job satisfaction

Lack of mentorship increased job stress and psychological disempowerment, limited women’s networking opportunities and detracted from job satisfaction by creating a sense of isolation, discontent and discouragement [35, 43, 44, 46].

#### Limited career development

Without adequate mentoring, career development, academic productivity and promotion were compromised [34, 38] and women were more likely to consider leaving academia [44, 46, 53].

#### Reduced academic productivity

The studies provide evidence indicating links between the lack of or inadequate mentoring and factors that disrupt or compromise academic productivity by limiting effective transition to the academic role, networking, academic craftsmanship and collaboration [38, 44, 46, 56].

### Gender issues in mentoring

More than half of the articles reviewed highlighted specific gender issues. Despite women more often considering mentoring more important than men [56], and demonstrating significantly greater improvement in professional development from mentoring [42], women were less likely to have mentors or to receive formal mentorship early in their career compared to men [43, 49].

Many studies reported not having enough senior women to mentor junior women [38, 45, 46, 53]. In general, women were more likely to be mentored by men [37, 43, 51, 54], although they preferred female mentors to seek better advice on career-life planning [61], work-life balance [51, 55], and timing of maternity leave [55]. Koopman and Thiedke [45] suggested that multiple mentors targeting career and lifestyle issues may suit female faculty.

While Wasserstein, et al. [58] found no significant differences in satisfaction with mentoring between those with a mentor of the same or different gender, Ramanan, et al. [50], considered gender issues in mentoring equally important for women and men.

## Discussion

This review has synthesized the research evidence about mentorship generated in the aftermath of corporatist changes in higher education, with the specific intent to identify the benefits, enablers and barriers to female health academics accessing suitable mentorship, the consequences of the lack of, or inadequate mentorship, and gaps in knowledge. The results, though somewhat limited and the evidence variable and context-bound, reinforce the value of mentoring female health academics as an ideal strategy for improving academic craftsmanship and productivity, promoting women’s career advancement, building female mentoring capacity and promoting retention, issues that resonate with the broader literature [1, 4, 16, 28].

The personal, professional and institutional benefits of mentoring women feature prominently in this review. The accrual of benefits over time are also congruent with the findings of others [1, 28] and augur well for advancing women’s academic careers. These beneficial outcomes of mentoring are of strategic value to the new knowledge economy which, like other social institutions, has become preoccupied with performance expectations, measured outputs and status [8, 62].

For the ‘craftsman’, the driving motive for performance is mastery [62] and the pursuit of excellence requires committed physical effort, skilled engagement and communal understanding of the tacit knowledge and skills required to produce excellent outcomes [62]. The notion of ‘craftsmanship’ has been applied widely, including in the health sciences [62, 63]. In the management sciences, the notion of ‘academic craftsmanship’ has been applied to denote ‘the noble and socially responsible pursuit of perfection in creating new understandings about the world of organizations’ (p. 1214) [64]. In this review, academic craftsmanship was considered as the mastery of skills associated with being a successful academic; that is, proficiency in teaching, research, manuscript and grant-writing, community service, strategic networking and collaboration, maintaining professional visibility, and ability to navigate higher education, manage difficult situations and negotiate desired outcomes. As such, the concept aligns well with outcomes that have been attributed to mentorship such as personal and professional development, academic productivity and becoming a successful academic [1].

Arguably, the notion of academic craftsmanship in the context of mentoring, fits like a hand in a glove, whereby the master academic craftsman, guides the development of a novice or junior craftsman, teaching them their craft somewhat like an apprentice; supporting, coaching, reviewing and refining their mastery of the role and promoting their status in the field [62]. However, it is salient to note that the threats to craftsmanship in the context of contemporary healthcare [62, 63], echo changes in higher education, particularly, the marginalisation and ‘invisibility’ of ‘peripheral’ sessional staff, often segregated from other staff and reliant on electronic media for guidance and connectivity [9].

Factors enabling mentoring of female health academics were the availability of a suitable mentor, mentor expertise, supportive relationships, mutuality and responsiveness to shifting needs. However, similar to our findings, others have found that women have limited access to suitable senior female mentors and that personal and relational factors compromise mentorship between men and women [2, 4]. Participants without mentors and those with unhelpful mentors sought alternatives through ‘trial and error’, attending nurse educators’ conferences, blog sites, watching experienced faculty teach and seeking informal mentors [44]. Changes resulting from corporatizing higher education, together with the ageing of the professoriate, have compounded this already fraught situation [4] and further limited the number of senior women faculty able and willing to mentor [2, 11, 16]. The unintended legacies of these changes in higher education have exacerbated the challenges of providing mentorship; especially for women who bear the brunt of casualization, work-intensification and conflicting role priorities [9, 14, 26].

Sambunjak, et al. [4] identified a lack of clarity about the importance of the mentor’s gender for women. The importance of females in academia being guided to manage work and family commitments has become particularly important in the context of casualization and work-intensification [1, 9]. Furthermore, a supportive, inclusive environment has been recognized to play an important part in addressing gender inequity [1, 65] and attrition [40, 46]. Flexible, voluntary and group mentoring models provide a solution that could enable female academics to find a mix of mentors able to address their multiple and evolving needs.

Collectively, the studies reviewed have reinforced the findings of others that a top-down approach to mentoring, whereby a mentee is assigned a senior mentor without regard for personal factors, may undermine outcomes [1, 18, 66]. The outcomes of this review emphasize the need for individual needs and preferences of the mentee to be considered and to adopt more flexible models of mentoring that account for the chemistry and complex interplay between mentors and mentees.

The structural barriers that block mentorship for female faculty reflect the higher education environment; overwhelming teaching loads, shortage of mentors and lack of time, institutional support and incentives [9, 56, 67]. Performance expectations in higher education have been increasingly geared to measured outputs [5, 8]. To ‘survive’, academics have needed to meet performance expectations and divest themselves of non-essential teaching and research demands [8]. Mentorship has not normally featured in workload allocations and has rarely been acknowledged by institutions [16, 66]. Though many of the organizational barriers identified in this review are not unique to women [3, 68], they are compounded by the lack of senior women available to provide mentorship. Promisingly, this review reveals a range of successful peer-supported, facilitated and co-mentoring models that could buffer the shortage of senior female mentors and the consequences of inadequate mentoring.

### Consequences of inadequate mentoring

This review found the consequences of inadequate mentoring to be isolation, disempowerment, job dissatisfaction, stress and limited career development; factors conducive to burnout and attrition [3, 11, 69]. These findings are neither unique to women, nor exclusive to health faculties [1, 70], however they reinforce the need for female health academics, especially early career academics, to have access to quality mentorship [4, 29, 71]. While there have been few empirical studies that testify to the effectiveness of mentoring in health academia [4, 19], there is a broad body of evidence that supports the need for mentorship and this review provides further evidence supporting recommendations to mainstream mentoring in medicine [4] and nursing [69, 72].

### Gaps in the literature

Despite the need to address gender inequities and career advancement, much of the literature on mentoring in the health sciences is generalised, the level of evidence weak and lacking in gender analysis. The gendered and work similarities between medical, nursing and other health academics suggest mentorship requires attention. Despite the prevalence of women in the health sciences, there were few studies in areas other than medicine that examined mentorship from a gendered perspective. This is an important oversight as female faculty often see themselves as ‘outsiders’ in the context of the academic workplace [40, 65], and regardless of clinical expertise, ill-prepared for the academic role [16, 28, 73].

Although Athanasiou, et al. [33] found few differences in results by gender they attributed the lack of significant differences in mentoring outcomes to the culture of the workplace and concerted efforts, policies and programs implemented to create a supportive culture conducive to promoting women’s academic success. This reference to culture and the environment signifies another gap in the literature, the limited attention given to the workplace culture, environment, valuing of mentorship and changing priorities. While some studies and reviews of mentorship acknowledge the organizational, personal and relational factors that impact mentoring outcomes, they have largely neglected the academic environment. Arguably, the organizational structures and culture typifying the global knowledge economy may have undermined job satisfaction and potentially, an academic’s ability and inclination to provide mentorship [2, 8, 56]. Similarly, although the restructuring of higher education has led to the integration of smaller institutions into large, multi-campus institutions, scant attention has been given to mentoring in the context of satellite or rural campuses. This is another important oversight because changes in staffing and role expectations of smaller satellite campuses compound the issues of access, availability and ‘fit’ of appropriate mentors.

Despite various models of mentoring being evaluated, no studies compared the gendered outcomes of different models of mentoring or the use of online mentoring. Most of the studies reviewed utilized cross-sectional, self-report designs with minimal use of validated instruments; factors which limited comparison across studies and precluded a meta-analysis. While six studies examined follow-up, outcomes ranging from 10 months to 6 years, only three studied the longer-term benefits of mentorship over four years or more. The dearth of longitudinal and high-level empirical studies that consider gender in the context of the contemporary higher education environment, continue to limit the evidence available about specific models of mentoring and their outcomes for women.

### Limitations and strength of evidence

This review was limited to studies of mentoring of women in health faculty, published in English in peer-reviewed journals from 2000 to 2018. Though we undertook a comprehensive search of four databases deemed most likely to elicit mentoring studies in faculties of health, it is possible some relevant studies may have been missed. As this was an integrative review and we sought to include all valuable informational content, we included all studies that met our inclusion criteria, and did not undertake quality ratings. The preponderance of data from academic medicine flags a bias in understanding yet highlights the priority given to mentoring as an investment in recruiting and retaining medical academics. While the lack of conceptual clarity around the notion of mentoring limited our ability to synthesize data, it yielded rich insight into new models and varied possibilities.

## Conclusion

This integrative review has synthesized what is known about the benefits, enablers and barriers to mentoring in the context of female academics in health faculties from 2000 to 2018, the effects of a lack of, or inadequate mentoring, and gaps in knowledge. In synthesising the evidence, this review provides a compelling case for institutions to invest in mentoring programs as a mechanism to support role transition, empower and retain new faculty and build female mentoring capacity. These results provide evidence that the provision of effective mentoring for female health academics is contingent on the organizational environment, specifically, workplace structures and relationships, and represents a long-term investment that can benefit academics and their mentors. Furthermore, mentorship assists higher education institutions to address faculty shortages, increase retention and productivity, advance female academics’ careers and build female mentoring capacity. The urgent need to address these issues necessitate that new strategies be adopted to capture the enablers and circumvent the personal, relational and organizational impediments to mentoring. This review highlights the need for further research on academic mentorship from a gendered and environmental perspective, particularly large scale empirical studies measuring comparative outcomes of different models of mentoring, and longitudinal studies into the value of mentoring and its potential to advance women’s academic careers.

## Supporting information

S1 Table Sample search strategy. (DOCX)

## References

[1] Gardiner M, Tiggemann M, Kearns H and Marshall K. Show me the money! An empirical analysis of mentoring outcomes for women in academia. Higher Education Research & Development. 2007; 26(4): 425–42.

[2] Kashiwagi DT, Varkey P and Cook DA. Mentoring programs for physicians in academic medicine: A systematic review. Academic Medicine. 2013; 88(7): 1029–37. 10.1097/ACM.0b013e318294f368 23702518

[3] Nick JM, Delahoyde TM, Prato DD, Mitchell C, Ortiz J, Ottley C, et al Best practices in academic mentoring: A model for excellence. Nursing Research & Practice. 2012: 1–9. 10.1155/2012/937906 PMC3366249 22685645

[4] Sambunjak D, Straus SE and Marušić A. A systematic review of qualitative research on the meaning and characteristics of mentoring in Academic Medicine. Journal of General Internal Medicine. 2010; 25(1): 72–78. 10.1007/s11606-009-1165-8 19924490 PMC2811592

[5] Houston D, Meyer LH and Paewai S. Academic staff workloads and job satisfaction: Expectations and values in academe. Journal of Higher Education Policy and Management. 2006; 28(1): 17–30.

[6] Neumann R and Guthrie J. The corporatization of research in Australian higher education. Critical Perspectives on Accounting. 2002; 13(5): 721–41.

[7] Pick D. The re‐framing of Australian higher education. Higher Education Quarterly. 2006; 60(3): 229–41.

[8] Guthrie J and Neumann R. Economic and non-financial performance indicators in universities: the establishment of a performance-driven system for Australian higher education. Public Management Review. 2007; 9(2): 231–52.

[9] Kimber M. The tenured'core'and the tenuous' periphery': the casualisation of academic work in Australian universities. Journal of Higher Education Policy and Management. 2003; 25(1): 41–50.

[10] Winter R and Sarros J. The academic work environment in Australian universities: a motivating place to work? Higher Education Research and Development. 2002; 21(3): 241–58.

[11] Dunham-Taylor J, Lynn CW, Moore P, McDaniel S and Walker JK. What goes around comes around: Improving faculty retention through more effective mentoring. Journal of Professional Nursing. 2008; 24(6): 337–46. 10.1016/j.profnurs.2007.10.013 19022206

[12] Nardi DA and Gyurko CC. The global nursing faculty shortage: Status and solutions for change. Journal of Nursing Scholarship. 2013; 45(3): 317–26. 10.1111/jnu.12030 23895289

[13] Reid TP, Hinderer KA, Jarosinski JM, Mister BJ and Seldomridge LA. Expert clinician to clinical teacher: Developing a faculty academy and mentoring initiative. Nurse Education in Practice. 2013; 13(4): 288–93. 10.1016/j.nepr.2013.03.022 23615037

[14] Baker M. Women graduates and the workplace: continuing challenges for academic women. Studies in Higher Education. 2016; 41(5): 887–900.

[15] Kena G, Hussar W, McFarland J, de Brey C, Musu-Gillette L, Wang X, et al. The Condition of Education 2016 (NCES 2016–144). 2016

[16] Sawatzky J-AV and Enns CL. A mentoring needs assessment: Validating mentorship in nursing education. Journal of Professional Nursing. 2009; 25(3): 145–50. 10.1016/j.profnurs.2009.01.003 19450785

[17] Department of Health. Health Workforce Data. Canberra: Australian Governement, Commonwealth of Australia; 2017. Available from: https://hwd.health.gov.au/publications.html#alliedh16

[18] Allen TD, Eby LT, Poteet ML, Lentz E and Lima L. Career benefits associated with mentoring for proteges: a meta-analysis. Journal of Applied Psychology. 2004; 89(1): 127–36. 10.1037/0021-9010.89.1.127 14769125

[19] Berk RA, Berg J, Mortimer R, Walton-Moss B and Yeo TP. Measuring the effectiveness of faculty mentoring relationships. Academic Medicine. 2005; 80(1): 66–71. 15618097 10.1097/00001888-200501000-00017

[20] Bierema LL and Merriam SB. E-mentoring: Using computer mediated communication to enhance the mentoring process. Innovative Higher Education. 2002; 26(3): 211–27.

[21] Thorpe K and Kalischuk RG. A collegial mentoring model for nurse educators. Nursing Forum. 2003; 38(1): 5–15. 12743968 10.1111/j.1744-6198.2003.tb01198.x

[22] Winter R. Academic manager or managed academic? Academic identity schisms in higher education. Journal of Higher Education Policy and Management. 2009; 31(2): 121–31.

[23] Peters MA and Boylston M. Mentoring adjunct faculty: Innovative solutions. Nurse Educator. 2006; 31(2): 61–64. 16601611 10.1097/00006223-200603000-00006

[24] Smith J and Zsohar H. Essentials of neophyte mentorship in relation to the faculty shortage. The Journal of Nursing Education. 2007; 46(4): 184–86. 17474489 10.3928/01484834-20070401-08

[25] Carr PL, Gunn CM, Kaplan SA, Raj A and Freund KM. Inadequate progress for women in academic medicine: findings from the National Faculty Study. Journal of Women's Health. 2015; 24(3): 190–99. 10.1089/jwh.2014.4848 25658907 PMC4363794

[26] Winchester H and Browning L. Gender equality in academia: a critical reflection. Journal of Higher Education Policy and Management. 2015; 37(3): 269–81.

[27] National Leauge for Nursing. Position Statement: Mentoring of Nurse Faculty. Nursing Education Perspectives. 2006; 27(2): 110–13. 16733976

[28] Specht JA. Mentoring relationships and the levels of role conflict and role ambiguity experienced by novice nursing faculty. Journal of Professional Nursing. 2013; 29(5): e25–e31. 10.1016/j.profnurs.2013.06.006 24075268

[29] Sambunjak D, Straus SE and Marušić A. Mentoring in academic medicine: a systematic review. JAMA. 2006; 296(9): 1103–15. 10.1001/jama.296.9.1103 16954490

[30] Whittemore R and Knafl K. The integrative review: updated methodology. Journal of Advanced Nursing. 2005; 52(5): 546–53. 10.1111/j.1365-2648.2005.03621.x 16268861

[31] Soares C, Hoga L, Peduzzi M, Sangaleti C, Yonekura T and Silva D. Integrative review: concepts and methods used in nursing. Revista da Escola de Enfermagem da USP. 2014; 48(2): 335–45. 10.1590/s0080-6234201400002000020 24918895

[32] Torraco R. Writing integrative literature reviews: Guidelines and examples. Human Resource Development Review. 2005; 4(3): 356–67.

[33] Athanasiou T, Patel V, Garas G, Ashrafian H, Shetty K, Sevdalis N, et al Mentoring perception and academic performance: an academic health science centre survey. Postgraduate Medical Journal. 2016; 92(1092): 581–86. 10.1136/postgradmedj-2016-134313 26994000

[34] Blood EA, Ullrich NJ, Hirshfeld-Becker DR, Seely EW, Connelly MT, Warfield CA, et al Academic women faculty: Are they finding the mentoring they need? Journal of Women's Health. 2012; 21(11): 1201–08. 10.1089/jwh.2012.3529 22906003

[35] Carapinha R, Ortiz-Walters R, McCracken CM, Hill EV and Reede JY. Variability in women faculty's preferences regarding mentor similarity: A multi-institution study in academic Medicine. Academic Medicine. 2016; 91(8): 1108–18. 10.1097/ACM.0000000000001284 27332871 PMC4965308

[36] Chung CE and Kowalski S. Job stress, mentoring, psychological empowerment, and job satisfaction among nursing faculty. Journal of Nursing Education. 2012; 51(7): 381–88. 10.3928/01484834-20120509-03 22588567

[37] Colletti LM, Mulholland MW and Sonnad SS. Perceived obstacles to career success for women in academic surgery. Archives of Surgery. 2000; 135(8): 972–77. 10922261 10.1001/archsurg.135.8.972

[38] De Saxe Zerden L, Ilinitch TL, Carlston R, Knutson D, Blesdoe BE and Howard MO. Social Work faculty development: An exploratory study of non-tenure-track women faculty. Journal of Social Work Education. 2015; 51(4): 738–53.

[39] Dutta R, Hawkes SL, Kuipers E, Guest D, Fear NT and Iversen AC. One year outcomes of a mentoring scheme for female academics: a pilot study at the Institute of Psychiatry, King's College London. BMC Medical Education. 2011; 11: 13 10.1186/1472-6920-11-13 21473749 PMC3094330

[40] Elliott BA, Dorscher J, Wirta A and Hill DL. Staying connected: Native American women faculty members on experiencing success. Academic Medicine. 2010; 85(4): 675–79. 10.1097/ACM.0b013e3181d28101 20354388

[41] Files JA, Blair JE, Mayer AP and Ko MG. Facilitated peer mentorship: A pilot program for academic advancement of female medical faculty. Journal of Women's Health. 2008; 17(6): 1009–15. 10.1089/jwh.2007.0647 18681821

[42] Fleming GM, Simmons JH, Xu M, Gesell SB, Brown RF, Cutrer WB, et al A facilitated peer mentoring program for junior faculty to promote professional development and peer networking. Academic Medicine. 2015; 90(6): 819–26. 10.1097/ACM.0000000000000705 25830537 PMC4446138

[43] Foster SW, McMurray JE, Linzer M, Leavitt JW, Rosenberg M and Carnes M. Results of a gender-climate and work-environment survey at a midwestern academic health center. Academic Medicine. 2000; 75(6): 653–60. 10875512 10.1097/00001888-200006000-00019

[44] Jeffers S and Mariani B. The effect of a formal mentoring program on career satisfaction and intent to stay in the faculty role for novice nurse faculty. Nursing Education Perspectives. 2017; 38(1): 18–22. 10.1097/01.NEP.0000000000000104 29194238

[45] Koopman RJ and Thiedke CC. Views of family medicine department Chairs about mentoring junior faculty. Medical Teacher. 2005; 27(8): 734–37. 10.1080/01421590500271209 16451897

[46] Levine RB, Lin F, Kern DE, Wright SM and Carrese J. Stories from early-career women physicians who have left academic medicine: A qualitative study at a single institution. Academic Medicine. 2011; 86(6): 752–58. 10.1097/ACM.0b013e318217e83b 21512363

[47] Mayer AP, Blair JE, Ko MG, Patel SI and Files JA. Long-term follow-up of a facilitated peer mentoring program. Medical Teacher. 2014; 36(3): 260–66. 10.3109/0142159X.2013.858111 24286367

[48] McGuire LK, Bergen MR and Polan ML. Career advancement for women faculty in a U.S. School of Medicine: Perceived needs. Academic Medicine. 2004; 79(4): 319–25. 15044163 10.1097/00001888-200404000-00007

[49] McMains KC, Rodriguez RG, Peel J, Yun HC, True MW and Jones WS. Assessing mentorship experiences of faculty at a military academic center: Challenge and opportunity. South Medical Journal. 2018; 111(5): 262–67. 10.14423/SMJ.0000000000000799 29767217

[50] Ramanan RA, Phillips RS, Davis RB, Silen W and Reede JY. Mentoring in medicine: Keys to satisfaction. American Journal of Medicine. 2002; 112(4): 336–41. 11893387 10.1016/s0002-9343(02)01032-x

[51] Seemann NM, Webster F, Holden HA, Moulton CA, Baxter N, Desjardins C et al Women in academic surgery: why is the playing field still not level? American Journal of Surgery. 2016; 211(2): 343–9. 10.1016/j.amjsurg.2015.08.036 26723836

[52] Simon CE, Bowles DD, King SW and Roff LL. Mentoring in the careers of African American women in social work education. Affilia. 2004; 19(2): 134–45.

[53] Sonnad SS and Colletti LM. Issues in the recruitment and success of women in academic surgery. Surgery. 2002; 132(2): 415–9. 12219043 10.1067/msy.2002.127694

[54] Steele MM, Fisman S and Davidson B. Mentoring and role models in recruitment and retention: a study of junior medical faculty perceptions. Medical Teacher. 2013; 35(5): e1130–e38. 10.3109/0142159X.2012.735382 23137243

[55] Straus SE, Chatur F and Taylor M. Issues in the mentor-mentee relationship in academic medicine: A qualitative study. Academic Medicine. 2009; 84(1): 135–39. 10.1097/ACM.0b013e31819301ab 19116493

[56] Turnbull BJ and Roberts K. Nurse-academics' mentorship: rhetoric or reality? Collegian. 2005; 12(2): 33–38. 16619911 10.1016/s1322-7696(08)60491-6

[57] Varkey P, Jatoi A, Williams A, Mayer A, Ko M, Files J, et al The positive impact of a facilitated peer mentoring program on academic skills of women faculty. BMC Medical Education. 2012; 12: 14 10.1186/1472-6920-12-14 22439908 PMC3325854

[58] Wasserstein AG, Quistberg DA and Shea JA. Mentoring at the University of Pennsylvania: Results of a Faculty Survey. Journal of General Internal Medicine. 2007; 22(2): 210–14. 10.1007/s11606-006-0051-x 17356988 PMC1824746

[59] Welch JL, Jimenez HL, Walthall J and Allen SE. The women in emergency medicine mentoring program: an innovative approach to mentoring. Journal of Graduate Medical Education. 2012; 4(3): 362–6. 10.4300/JGME-D-11-00267.1 23997883 PMC3444192

[60] Seritan AL, Bhangoo R, Garma S, Dubé J, Park JH and Hales R. Society for women in academic psychiatry: a peer mentoring approach. Academic Psychiatry. 2007; 31(5): 363–66. 10.1176/appi.ap.31.5.363 17875620

[61] Alisic S, Boet S, Sutherland S and Bould MD. A qualitative study exploring mentorship in anesthesiology: perspectives from both sides of the relationship. Canadian Journal of Anesthesia. 2016; 63(7): 851–61. 10.1007/s12630-016-0649-3 27060087

[62] Sennett R. The craftsman. Newhaven: Yale University Press, 2008.

[63] Coeckelbergh M. E-care as craftsmanship: virtuous work, skilled engagement, and information technology in health care. Medicine, Health Care and Philosophy. 2013; 16(4): 807–16. 10.1007/s11019-013-9463-7 23338289

[64] Baer M and Shaw J. Falling in love again with what we do: academic craftsmanship in the management sciences. Acad Manage J. 2017; 60: 1213–1217.

[65] Gibson SK. Mentoring of women faculty: The role of organizational politics and culture. Innovative Higher Education. 2006; 31(1): 63–79.

[66] Bell A and Treleaven L. Looking for Professor Right: mentee selection of mentors in a formal mentoring program. Journal of Higher Education. 2011; 61(5): 545–61.

[67] Morrison LJ, Lorens E, Bandiera G, Liles WC, Lee L, Hyland R, et al Impact of a formal mentoring program on academic promotion of Department of Medicine faculty: A comparative study. Medical Teacher. 2014; 36(7): 608–14. 10.3109/0142159X.2014.899683 24804918

[68] Allen TD, Eby LT, O’Brien KE and Lentz E. The state of mentoring research: A qualitative review of current research methods and future research implications. Journal of Vocational Behavior. 2008; 73(3): 343–57.

[69] Nowell L, Norris JM, Mrklas K and White DE. Mixed methods systematic review exploring mentorship outcomes in nursing academia. Journal of Advanced Nursing. 2017; 73(3): 527–44. 10.1111/jan.13152 27650412

[70] Hart J. Dissecting a gendered organization: Implications for career trajectories for mid-career faculty women in STEM. Journal of Higher Education. 2016; 87(5): 605–34.

[71] Paul S, Stein F, Ottenbacher KJ and Liu Y. The role of mentoring on research productivity among occupational therapy faculty. Occupational Therapy International. 2002; 9(1): 24–40. 12375006 10.1002/oti.154

[72] Gruber-Page M. The value of mentoring in nursing: An honor and a gift. Oncology Nursing Forum. 2016; 43(4): 420–22. 10.1188/16.ONF.420-422 27314184

[73] Frantz JM, Rhoda A, Rowe M, Phillips J, Karachi F, Mlenzana N, et al Mentoring and coaching in promoting publications in the Department of Physiotherapy at a local university in South Africa. South African Journal of Physiotherapy. 2010; 66(2): 35–41.

