# Supplementary material for: Benefits, barriers and enablers of mentoring female health academics: An integrative review
Source: PLoS One. 2019 Apr 18;14(4):e0215319. doi: 10.1371/journal.pone.0215319 (PMC6472752; doi:10.1371/journal.pone.0215319)
Supplement: S1 Table — (DOCX) [file pone.0215319.s001.docx]

**CINAHL (Cumulative Index to Nursing and Allied Health Literature)**

| (MH women OR TI women OR AB women OR TI woman OR AB woman OR TI female OR AB female ) AND ( MH mentorship OR TI mentor* OR AB mentor* OR TI mentee* OR AB mentee* OR TI protege* OR AB protege*) AND ( MH Colleges and Universities OR TI university OR AB university OR TI universities OR AB universities OR TI faculty OR AB faculty OR TI academi* OR AB academi* OR TI higher education OR AB higher education ) NOT student* NOT patient* NOT pre-registration NOT new graduate* NOT supervis* NOT preceptor* NOT baccalaureate NOT undergraduate NOT leader |
| --- |
